# Potential Toxic Metal Concentration and Risk Assessment in Agricultural Soil and Lentil Crop (*Lens culinaris Medik*) in Dawunt Woreda, Northwest Wollo, Ethiopia

**DOI:** 10.1155/2024/8985402

**Published:** 2024-04-12

**Authors:** Baynesagn Biyazen Belay, Asamene Embiale Taye

**Affiliations:** Department of Chemistry, College of Natural and Computational Science, Woldia University, P.O. Box 400, Woldia, Ethiopia

## Abstract

Health implications for the population due to consuming contaminated crops have been a great concern worldwide. This study aimed to measure the levels of potential toxic elements in lentils and their growing soil in Dawunt Woreda, Ethiopia. Accordingly, 15 soil samples along with the lentil samples were collected to measure the level of potential toxic elements, including chromium (Cr), manganese (Mn), iron (Fe), lead (Pb), cadmium (Cd), copper (Cu), and cobalt (Co), by using an inductively coupled plasma optical emission spectrometer and for assessing the potential ecological and human health risk. The wet digestion method using aqua regia (HCl/HNO_3_ 3 : 1) was employed for soil and lentil sample preparation. The mean concentrations of Fe, Mn, Co, Cu, Cd, Pb, and Cr in the lentil sample were 60.4, 9.68, 0.75, 5.7, 0.25, 0.9, and 1.15 mg/kg, respectively. In soil, the mean concentrations of Fe, Mn, Co, Cu, Cd, Pb, and Cr were 649, 19.9, 3.32, 40.0, 15.2, 1.83, and 69.1 mg/kg, respectively. All of the potential toxic metals in agricultural soil and lentil samples were found to be below the reference level set by the World Health Organization, except Cd, in the soil samples. Five single metal and three cumulative pollution index parameters were employed for the data and results showed that Fe, Cu, and Cr moderately pollute the soil and are highly contaminated by Cd. The cumulative pollution indices also confirmed that the extent of soil pollution varied from highly contaminated to moderate contamination. The possible health risks at various exposure routes have also been estimated. The single-metal and cumulative-metals health risks (cancer and noncancer) of adults and children due to chronic exposure to soil and consumption of lentils were estimated using the health quotient and health index values as per the United States Environmental Protection Agency guidelines. Thus, the results revealed no significant adverse health risks (cancer and noncancer) for adults and children. Therefore, the inhabitants in the study area have no significant health impacts due to either the consumption of lentil crops or exposure to agricultural soil particles.

## 1. Introduction

Environmental pollution, which is today viewed as a serious issue, is mainly due to population increase, urbanization, industrialization, and transportation [1, 2]. Environmental pollution by potential toxic metals (PTEs) and associated food safety is a menace around the globe. Among the environment segment, soil can bind various chemicals and, therefore, can be considered an important holding for contaminants that may originate from different sources [3–5]. The soil contamination by PTEs has exerted long-term ecological and health risks. Crops cultivated in contaminated agricultural soils have a high tendency to accumulate PTEs and may cause severe carcinogenic and noncarcinogenic risks to the human body [6, 7]. In agricultural areas, PTEs can get into the soil through either anthropogenic sources including industrial effluents, domestic sewage, excessive use of phosphate fertilizers, herbicides, pesticides, road construction, road maintenance, and vehicle exhaust emissions, or natural sources including wild forest firing, volcanic activities, rock weathering, rainwater, and atmospheric deposition [8–14]. Hence, it is paramount to know the status of PTEs' pollution level in agricultural soil using the developed methods (contamination factor, pollution load index, geoaccumulation index, degree of contamination, and others) for a comprehensive understanding of human risk. In this regard, many studies related to ecological and health risk assessment of PTEs due to consumption of crops and exposure to its growing agricultural soil particles have been reported in different parties of the world [7, 15–19].

The exposure to soil particles during tilling and consumption of agriculture products cultivated in PTEs-polluted soil can pose several diseases due to environmental persistence, bioaccumulation, and biomagnification character of PTEs. The level of PTE in the plant mainly depends on the plant species and genetic characteristics, concentration, and bioavailability of metal and soil physicochemical properties including pH, salinity, and organic matter of soil. PTEs can enter human bodies through all three exposure routes (oral, dermal contact, and inhalation) [15, 20, 21]. Metals such as Zn, Fe, Mn, Cu, and Ni are essential for biological systems in the human body, acting as structural and catalytic components of proteins and enzymes. However, they are the cause of cancers, changes in bones, digestive disorders, and even death, when they are present in large amounts [12, 22, 23]. On the other hand, metals such as Cd and Pb have many adverse effects on humans including damage and dysfunction of the liver, chromosomal abnormalities, and cancer even at low concentrations [12, 23]. Chronic and acute exposure to PTEs causes different adverse effects, including hypertension, lung damage, anaemia, diabetes, hypoglycemia, arthritis, kidney disease, osteoporosis, cancer, acne, autism, hair loss, depression, elevated cholesterol, infections, liver dysfunction, tooth decay, vitamin C deficiencies, and others [12, 14, 21, 24]. Consequently, monitoring and control of pollution sources and contaminant levels of PTEs in the environment is required to minimize their impact on the ecosystem [13, 25]. In this regard, the risk analysis technique is used for quantifying and estimating the present and future levels of risk. The risk assessment of humans, which is part of risk analysis involves scientific hazard identification, dose-response analysis, exposure assessment, and risk characterization steps. The information related to the type of hazardous substance, exposure duration, exposure frequency, and dose is needed. The risk assessment associated with exposure to PTEs' includes carcinogenic and noncarcinogenic assessments [5, 21, 23].

Ethiopia is one of the top nine lentil-producing countries in the world, where the total cultivated amount is about 800 kg/hectares. Lentil crop has been a very crucial crop for human and animal nutrition for over 8, 000 years, as well as maintaining and improving soil fertility [26, 27]. Dawunt woreda is one of the largest lentil-producing areas in Ethiopia. The economy of the inhabitants mainly depends on cultivation, where most of the families are involved in farming throughout the day. The farmers in the study area have removed weeds by hand during previous times. However, nowadays, they are using different types of herbicides and pesticides to kill the weeds and pests, respectively, that tend to increase PTEs accumulation in the farmlands. In addition, the farmers of the province also use enormous amounts of fertilizers to enhance the yield of crops which can also further contaminate the agricultural soil with PTEs [11, 28, 29]. As a result, any crop cultivated in the area might have a high contamination probability with PTEs that are potentially harmful to inhabitants [30, 31]. Therefore, it is essential to measure the level of contaminants in food products (lentil crop, a typical cereal and the primary food used by the local community) and its growing soil to know their hazard level. In addition, there are limited data related to PTE levels in lentil crops and agricultural soil in Ethiopia, which leads to inadequate assessment of the actual risk assessment of PTEs' exposure from crops. Consequently, this study aims to investigate (1) the levels of selected PTEs in lentil crops (*Lens culinaris Medik*) and its growing agricultural soil at Dawunt Woreda, Northwest Wollo, Ethiopia, (2) the health risk of the investigated PTEs as per the United State Environmental protection Agency (USEPA), and (3) the soil pollution level using different parameters such as contamination factor, pollution load index, geoaccumulation index, degree of contamination, and others.

## 2. Materials and Methods

### 2.1. Description of the Study Area

**Figure 1:**
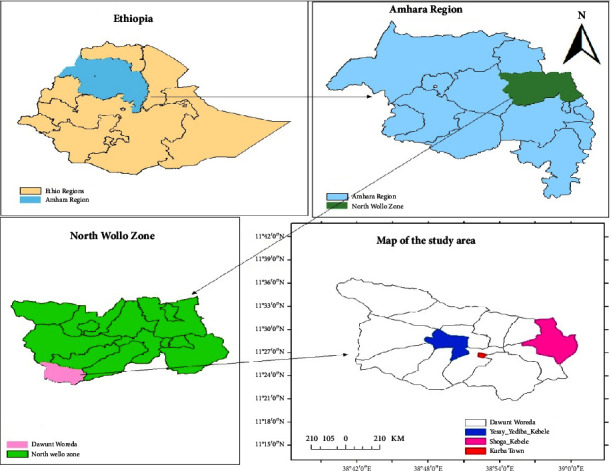
Map of the study area (drawn by ArcGIS software).

Dawunt woreda is the agricultural area in North Wollo Zone, Amhara region, Ethiopia, where the total population of the woreda is about 81826 (40849 males and 40430 females). The main livelihood for the farmers in the study province (Dawunt woreda) is dependent on rain-fed agriculture. Over time, continuous farming depletes nutrients in soil and as a result, large amounts of nitrogenous and phosphate fertilizers are used by farmers. The administrative center of this woreda is Kurba town, which is located 352 km from Bahir Dar city (capital of Amhara regional state), 192 km from Woldia town (the main town of North Wollo administrative zone), and 593 km northwest of Addis Ababa. Astronomically, the woreda is located between 11°20′0″N-11°36′30″ N latitude and 38033′30″E-39°0′30″ E longitude. The average annual rainfall of the woreda is 600 mm, with high variability between 250 mm and 950 mm. The mean annual temperature is approximately 20°C [32]. The geographical map of the present study area is given in Figure 1.

### 2.2. Sample Collection and Digestion

The sampling locations for soil and lentil are Yesay-yediba, Shoga, and Kurba kebele, which are known for large lentil crop production in the Woreda. All plastic containers used for sampling were washed thoroughly with liquid soap, rinsed with deionized water, and then soaked in 10% HNO_3_ solution for 24 hours before being taken to the field for sampling. The reagents and chemicals used in this study include HNO_3_ (69%, Merck, France), 37% HCl (Fine Chem. Industries, Mumbai, India), HClO_4_ (Fine Chem. Industries, Mumbai, India), and 30% H_2_O_2_ (Scharlau, European Union).

### 2.3. Sampling of Soil and Lentil Samples

The soil samples (7.5 kg; 500 g from fifteen different points from the three kebeles) were collected using purposive and random sampling techniques (by considering the topography and area of the farm) in June 2022. The soil samples were collected from topsoil at depths of 0–20 cm by using a soil auger, which represents the plough layer and the average root zone for nutrient uptake and contains excessive PTEs that burden lentil plants [33, 34]. The collected samples were mixed thoroughly into one composite sample to obtain a representative sample of 1 kg. The sample was placed in a polythene bag, sealed and labeled appropriately, and then transported to the laboratory. On the other hand, after identifying to which farmer the farmlands belong, the lentil seeds were sampled from local farmers at the household level, which minimized the error during enrichment and contamination factor calculations. Hence, about 300 g of lentil samples from fifteen farmers' households were collected and combined to form a composited sample. After the composite samples were made, about 500 g of the lentil sample was brought to the laboratory, and then the samples were washed with distilled water and rinsed several times with deionized water. It was then dried in an oven at 70°C for 24 hours. After cooling, the samples were ground to a fine powder and packed in clean, labeled, decontaminated plastic containers for further digestion.

### 2.4. Digestion of Soil and Lentil Sample

The soil sample was dried in the open air for one week, crushed, and sieved mechanically using a 1.0 mm sieve. After which, 1.25 g of the sample was digested with 20 mL aqua regia (HCl/HNO_3_ 3 : 1) in a beaker (open-beaker digestion) on a controlled plate. The mixed solution was heated to near dryness and cooled to ambient temperature. Then, 5.0 mL of H_2_O_2_ was added in parts to complete the digestion, and the resulting mixture was heated again to dryness in a fume cupboard. The beaker walls were rinsed with 10 mL of deionized water, and 5 mL of HCl was added, mixed, and heated again. The resulting digest was allowed to cool, transferred to a 5 mL standard flask, and made up to the mark with distilled water. Pb, Cd, Cu, Co, Cr, Mn, and Fe, the potential toxic metal elements, were then analyzed by direct aspiration of the sample solution into inductively coupled plasma optical emission spectrometry (ICP-OES, (Model: ARCOS FHS12, USA)) with a detection range of 0.001 to 0.007 mg/kg [35].

Five grams of lentil seeds flour was weighed and transferred to a clean crucible, and the dry-ashing process was carried out in a muffle furnace by a stepwise increase in the temperature up to 550°C and left to ash at this temperature for 6 hrs. The sample was removed from the furnace, cooled, and transferred into a conical flask. The ash was wetted with water, and 2.5 mL of concentrated HNO_3_ was then added. The conical flask with a glass cover was intact and placed on a warm hot plate to commence wet ashing. The digestion was performed at 90 to 95°C for 1 hr. The ash was dissolved in 5 mL of 9.25% HCl and digested again on a hot plate until the white fumes ceased to exist and the sample reached 2 mL. Then, 2 mL of 70% HClO_4_ was added to the cooled solution, and heating was resumed until a clear solution appeared. As all HNO_3_ eventually evaporated, fumes of HClO_4_ appeared; heating was maintained until ashing was completed. The HClO_4_ was then removed by evaporation. The residue was treated with 5 mL of concentrated HCl, and the acid was refluxed in the beaker; an equal volume of water was then added with subsequent evaporation to dryness and this refluxing process with concentrated HCl followed by evaporation to dryness was repeated. Finally, 1.0 mL of concentrated HCl was added, and the mixture was warmed. In brief, then, 15 mL of water was added, and the solution was heated for about 15 min. After cooling, 20 mL of distilled water was added and filtered using Whitman's filter. The filtered sample was diluted to the 50 mL standard volumetric flask mark and transferred to a 50 mL polyethylene storage bottle until analysis. All the samples and blanks were digested in triplicate [36, 37].

### 2.5. Calibration of ICP-OES and Method Validation

Calibration curves for Fe, Cd, Mn, Pb, Cr, Co, and Cu were obtained using suitable standard solutions prepared from stock solutions. The calibration curve for the present study showed a good linear range with regression coefficients (*R*^2^) ≥ 0.9945. The series of standard solutions, correlation coefficient, and the calibration equations for each metal are given in supplementary Table S1. To validate the analytical method, the method detection limit (MDL), the limit of quantification (LOQ), the percent relative standard deviation (RSD), and the recovery test were carried out [38]. For the present study, the MDL and LOQ were obtained using equations 1 and 2, respectively, provided in supplementary Table S2, where the triplicate analysis of five blank samples was digested with the same digestion procedure as the actual samples to get the standard deviation of the mean reagent empty signal [30, 31]. The corresponding MDL and LOQ values for the current study were found in the range of 0.003 to 0.175 and 0.01 to 0.57 mg/kg, and the values for each metal are provided in supplementary Table S2.

The method's reliability for investigated PTEs was assured by spiking the samples with a standard solution of known concentration of the target analytes (the recovery test). In contrast, the precision method was cheeked by RSD calculation. Percent recovery test and RSD were then calculated using equations 3 and 4, respectively, given in Supplementary Table 3. In this work, the percentage recoveries of PTE obtained for soil and lentil samples were 83–100% and 88–98%, which is within the acceptable range (80 to 120%). The percent RSD for both soil and lentils was also found in an acceptable range (<10%) [38]. The %RSD results and recovery test for each study are given in Supplementary Table 3.

### 2.6. Ecological Risk Assessment

Different types of single (contamination factor, geoaccumulation, and single risk index) and integrated (degree of contamination, modified degree of contamination, potential ecological risk index, pollution load index, and Nemerow pollution index) pollution indices have been applied for ecological risk assessment [2, 39–42]. Thus, contamination factor, geoaccumulation, single risk index, degree of contamination, modified degree of contamination, potential ecological risk index, pollution load index, and Nemerow pollution index are calculated by using enrichment factor equations (1)–(9), respectively.

Contamination factor (*C*_fi_): it describes a given toxic substance contamination and is calculated by using the following equation [39]:

(1) $$\begin{array}{c} C_{\text{fi}}=\frac{C_{i}}{C_{\text{iB}}}, \end{array}$$

where *C*_*i*_ is the concentration of the element in substrate samples and *C*_iB_ is the reference value (world average background values) of the element, and the values for each study metal are given in Table 1 [43, 44]. According to Hakanson (1980), contamination factors can be classified as follows: *C*_*fi*_ < 1, low contamination factor; 1 ≤ *C*_*fi*_ < 3, moderate contamination factor; 3 ≤ *C*_*fi*_ < 6, considerable contamination factor; and *C*_*fi*_ ≥ 6, very high contamination factor.

Geoaccumulation index (*I*_geo_): it is used to assess the intensity of anthropogenic contaminant deposition on the surface soil and is calculated by using the following equation [39, 44]:

(2) $$\begin{array}{c} I_{\text{geo}}=\log_{2}\left(\frac{C_{i}}{{1.5C}_{\text{ib}}}\right), \end{array}$$

where *C*_*i*_ is the element of interest concentration, while *C*_*iB*_ is the geochemical background value. Constant 1.5 allows analyzing natural fluctuations in the content of a given substance in the environment and detects very small anthropogenic influences. The pollution degree can be classified as follows: *I*_geo_ < 0, unpolluted; 0 < *I*_geo_ < 1, unpolluted to moderately polluted; 1 < *I*_geo_ ≤ 2, moderately polluted; 2 < *I*_geo_ ≤ 3, moderately to strongly polluted; 3 < *I*_geo_ ≤ 4, strongly polluted; 4 < *I*_geo_ ≤ 5, strongly to extremely polluted; and *I*_geo_ > 5, extremely polluted.

Single risk index (*E*_*r*_^*i*^): it quantitatively expresses the potential ecological risk of a given contaminant and is calculated by using the following equation [39]:

(3) $$\begin{array}{c} E_{r}^{i}=T_{r}^{i}\;x\;C_{\text{fi}}, \end{array}$$

where *C*_*fi*_ is the contamination factor and *T*_*r*_^*i*^ is the toxic response factor for the given substance. The *T*_*r*_^*i*^ values used were those provided by Hakanson (Mn = 10; Co = 5; Cu = 5; Pb = 5). The degree of contamination can be classified as follows: *E*_*r*_^*i*^ < 30, low contamination; 30 ≤ *E*_*r*_^*i*^ < 60, medium contamination; 60 ≤ *E*_*r*_^*i*^ < 120, high contamination; 120 ≤ *E*_*r*_^*i*^ < 240, very high contamination; and *E*_*r*_^*i*^ ≥ 240, extremely high contamination.

Degree of contamination (*C*_*D*_): it is defined as the sum of all contamination factors (*C*_fi_) for various potential toxic metals and is calculated by using the following equation [39]:

(4) $$\begin{array}{c} C_{D}=\overset{n}{\underset{i=1}{\sum }}C_{\text{fi}}, \end{array}$$

where *n* is the number of HM species. The contamination level is classified as follows: *C*_*D*_ < *n*, low contamination; *n* < *C*_*D*_ < 2*n*, moderate contamination; 2*n* < *C*_*D*_ < 4*n*, considerable contamination; and *C*_*D*_ > 4*n*, very high contamination.

Modified degree of contamination (*mCd*): it is defined as the sum of all contamination factors (*C*_fi_) for a given set of pollutants divided by the number of analyzed pollutants (*n*) and is given by the following equation [39]:

(5) $$\begin{array}{c} \text{mCd}=\frac{\sum_{i=1}^{n}C_{n}}{n}, \end{array}$$

where the value of mCd is 1.5 ≤ mCd ≤ 2, indicating low contamination; 2 < mCd ≤ 4, indicating moderate contamination; 4 < mCd ≤ 8, indicating high contamination; 8 < mCd ≤ 16, indicating very high contamination; 16 < mCd ≤ 32, indicating extremely high contamination; and mCd > 32, indicating super contamination.

Potential ecological risk index (RI): it is used to quantitatively express the potential risk of metals measured in the soil and is calculated by using the following equation [39]:

(6) $$\begin{array}{c} \text{RI}=\overset{n}{\underset{i=1}{\sum }}E_{r}^{i}=\overset{n}{\underset{i}{\sum }}T_{r}^{i}\;x\;C_{\text{fi}}, \end{array}$$

where *E*_*r*_^*i*^ is the ecological risk factor of the individual potential toxic metal. The toxicity level is classified as follows: RI < 150, low toxicity; 150 ≤ RI < 300, medium toxicity; 300 ≤ RI < 600, considerable toxicity; and RI ≥ 600, extremely high toxicity.

Pollution load index (PLI): PLI is a measure of the degree of overall contamination on a sampling site and provides simple but comparative means for assessing a site quality and is calculated by using the following equation [39, 44]:

(7) $$\begin{array}{c} \text{PLI}=\sqrt[{n}]{\overset{n}{\underset{i=1}{\prod }}C_{\text{fi}}}, \end{array}$$

where *n* is the number of metals analyzed and *C*_fi_ is the contamination factor. The pollution level is classified as follows: PLI < 1 no pollution, PLI = 1 baseline pollutant level present, and PLI > 1 heavy pollution.

Nemerow pollution index (PINemerow): it indicates the sediment quality. The index is similar to the modified degree of contamination index because it uses the average contamination factors of a suite of elements and is calculated by using the following equation. However, it also considers an element contamination impact using the maximum contamination factor to develop a weighted average.

(8) $$\begin{array}{c} {\text{PI}}_{\text{Nererow}}=\sqrt{\frac{{\left(1/n\sum_{i=1}^{n}C_{\text{fi}}\right)}^{2}+C_{\text{fi},\max}^{2}}{n}}, \end{array}$$

where *C*_(*fi*), _(max) is the maximum value of the single contamination factor of all potential toxic metals. The pollution level is classified as follows: PI_Nemerow_ < 0.7, safety domain; 0.7 ≤ PI_Nemerow_ < 1, warning limit; 1≤PI_Nemerow_ < 2, slight pollution; 2 ≤ PI_Nemerow_ < 3, moderate pollution; and PI_Nemerow_ > 3, heavy pollution.

Enrichment factor (EF): it is used to identify the degree of contamination of soil and possible sources of contamination (human-made or natural) and is estimated by using the following equation:

(9) $$\begin{array}{c} \text{EF}=\frac{{\left(C_{i}/C_{\text{Fe}}\right)}_{\text{sample}}}{{\left(C_{i}/C_{\text{Fe}}\right)}_{\text{background}}}, \end{array}$$

where C_Fe_ and *C*_*i*_ are the concentrations of iron and the PTEs (mg/kg) in soil samples and background soil samples. In this study, the average concentration of world soil was used as a reference metal for normalization. The soil pollution level is classified as extremely severe enrichment (EF > 50), very severe enrichment (25 ≤ EF < 50), severe enrichment (10 ≤ EF < 25), moderately severe enrichment (5 ≤ EF < 10), moderate enrichment (3 ≤ EF < 5), minor enrichment (1 ≤ EF < 3), and no enrichment (EF < 1) [24].

### 2.7. Transfer Factor (TF)

The transfer factor indicates the potential transfer of hazardous contaminants such as PTEs from the soil to the edible part of the crops. TF value that is significantly higher than one indicates the high capacity of the plant to absorb metals from the agricultural soil. The TF for each potential toxic metal was estimated by using the following equation [45, 46]:

(10) $$\begin{array}{c} \text{TF}=\frac{C_{\text{lent}}}{C_{\text{soil}}}, \end{array}$$

where *C*_lent_ and *C*_soil_ represent the HM concentration (mg/kg) in lentils and agricultural soil on dry weight bases.

### 2.8. Health Risk Assessment Method

This study employed the noncancer and cancer health risk assessment model established by USEPA. The PTEs in soil samples can be exposed to humans through direct ingestion of soil particles, inhalation of soil particles from the air, dermal contact with soil particles, and diet through the food chain (lentil in this study). The estimated average daily intake of PTEs soil and lentil samples was calculated by using the following equations [47, 48]:

(11) $$\begin{array}{c} {\text{ADI}}_{\text{ing}}=\frac{C_{s}\;x\;{\text{Ing}}_{R}\;x\text{ EF }x\text{ ED }x\text{ CF }}{\text{BW }x\text{ AT}}, \end{array}$$

(12) $$\begin{array}{c} {\text{ADI}}_{\text{inh}}=\frac{C_{s}\;x\;{\text{Inh}}_{R}\;x\text{ EF }x\text{ ED }}{\text{PEF }x\text{ BW }x\text{ AT}}, \end{array}$$

(13) $$\begin{array}{c} {\text{ADI}}_{\text{der}}=\frac{C_{s}\;x\text{ SA }x\text{ AF }x\text{ ABS }x\text{ EF }x\text{ ED }x\text{ CF }}{\text{BW }x\text{ AT}}, \end{array}$$

(14) $$\begin{array}{c} {\text{ADI}}_{\text{len}}=\frac{C_{\text{lent}}\;x\;{\text{Ing}}_{\text{lent}}\;x\text{ EF }x\text{ ED }x\text{ CF }}{\text{BW }x\text{ AT}}, \end{array}$$

where ADI_ing_ = average daily intake through ingestion (mg/kg-day), ADI_inh_ = average daily intake through inhalation (mg/kg-day), *C*_*s*_ = HM concentration in soil (mg/kg), *C*_lent_ = HM concentration in lentil (mg/kg), Ing_R_ = ingestion rate of soil 100 mg/kg for adults and 200 mg/kg for children, Ing_lent_ = ingestion rate of lentil (since there were no data available for Ing_lent_ of lentils for Ethiopian adults and children, this study used site-specific professional judgment that the average daily consumption of lentils for adults is 300 g/person-day and 100 g/person-day for children), EF = exposure frequency (350 days/year), ED = exposure duration (30 years for adults and 6 years for children), and CF = units conversion factor, 10^−6^ kg/mg. SA = exposure skin area (5700 and 2800 ^cm2^ for adults and children, respectively) AF = adherence factor (0.07 and 0.02 mg^·cm−2^ for adults and children, respectively), ABS = dermal absorption fraction (0.01 for both age groups), BW = body weight (60.7 kg for adults and 15 kg for children, site-specific), AT = averaging time (365 × ED for noncarcinogens and 65 × 365 for cancer risk), and Inh_R_ = inhalation rate (20 m^3^/day for adults and 7.6 m^3^/day for children) [1, 8, 49, 50].

#### 2.8.1. Noncancer Risk Assessment

The noncancer risk due to PTEs was assessed using the hazard quotient (HQ) and hazard index (HI) parameters. Thus, HQ can assess the single-metal noncancer risk, while HI can be used for multimetal noncancer risk. The values of HQ and HI were determined using equations (15) and (16), respectively. The calculations considered the average daily intake of PTEs through inhalation, ingestion, and dermal contact with soil and lentils [6].

(15) $$\begin{array}{c} \text{HQ}=\frac{\text{ADI}}{\text{RFD}}, \end{array}$$

(16) $$\begin{array}{c} \text{HI}=\overset{n}{\underset{K=1}{\sum }}\text{HQ}, \end{array}$$

where HQ is the target hazard quotient, ADI is the chronic daily intake of potential toxic metal (mg/kg), and RFD is the reference dose (mg/kg/day). If HQ or HI is higher than unity (HQ or HI > 1), there will be a severe health hazard to humans, whereas there will be no severe human health effects (HQ or HI < 1) [49, 51]. The RFD values of Fe, Cu, Mn, Pb, Cr, Cd, and Co for inhalation exposure are 2.2*E* − 04, 0.0402, 1.43*E* − 04, 352*E* − 03, 2.86*E* − 04, 0.001, and 5.71*E* − 06 mg/kg-day, respectively. The corresponding values of RFD for ingestion and dermal contact for Fe, Cu, Mn, Pb, Cr, Cd, and Co are 0.84, 0.04, 0.047, 0.035, 0.003, 001, and 0.02 and 0.07, 0.012, 1.84*E* − 03, 5.25*E* − 04, 5*E* − 05, 1*E* − 04, and 0.016 mg/kg-day [6, 52].

#### 2.8.2. Cancer Risk Assessment

The cancer risk of PTEs was assessed by single-metal cancer risk (*CR*_*k*_) and multimetal cancer risk (total cancer risk, TCR) and calculated by using the following equations:

(17) $$\begin{array}{c} {\text{CR}}_{k}=\text{ADI }x\text{ CSF}, \end{array}$$

(18) $$\begin{array}{c} \text{TCR}=\overset{n}{\underset{k=1}{\sum }}{\text{CR}}_{k}, \end{array}$$

where CR is the cancer risk over a lifetime by individual potential toxic metal ingestion, TCR is the total cancer risk over a lifetime by multiple metals, *CR*_*k*_ is the cancer risk of metal *k*, and CSF is the cancer slope factor (mg/kg-day) and the CSF for Pb, Cr and Cd for inhalation are 1*E* − 04, 0.084, and 1.8*E* − 03, respectively. 0.28, 0.5, and 0.64 were used for ingestion and dermal contact exposure routes. Hence, according to USEPA guidelines, if the values of CR or TCR are greater than 10^−4^, it indicates a high probability of an individual for developing the cancer risk over a lifetime; if CR or TCR is lower than 10^−6^, it is relatively safe; and if CR or TCR value is between 10^−4^ and 10^−6^, it is considered as the tolerable range [6, 49].

### 2.9. Statistical Analysis

The data from soil and lentil samples was subjected to statistical analysis using Pearson correlation and hierarchical cluster analysis (HCA) to estimate the origin of PTE sources [53]. Since, the variance of obtained data showed homogeneity, the ANOVA (analysis of variance) parametric test was employed. The mean concentration of the PTEs in the soil and lentil samples was compared using a one-sample *t*-test. The test was also applied for the mean concentration of the investigated PTEs compared with WHO/FAO maximum threshold values. Mean, standard deviation, and percent recovery were calculated using SPSS software version 20 and Microsoft Excel 2016 [21, 23].

## 3. Results and Discussion

### 3.1. Levels of Selected Potential Toxic PTEs in Soil and Lentil Samples

The results of the average concentration of Cd, Cu, Co, Pb, Mn, Fe, and Cr in soil and lentil samples from Dawunt Woreda farmlands are given in Table 1. The mean concentration of PTEs in soil samples decreased in the order of Fe > Cr > Cu > Mn > Cd > Co > Pb with the range value of 649–1.833 mg/kg. The highest level of Fe among the PTEs might be its abundance because iron is the fourth most abundant element in the earth's crust. The mean concentration value of all investigated PTEs (except Cd) in the soil revealed that they were lower than the WHO/FAO permissive limits. Even though most of the studied PTE concentrations fell below the critical permissible concentration level, they have a persistent nature in the soils that may lead to increased bioaccumulation in the plants [54].

The mean concentration of the studied PTEs was compared with other studies conducted in Ethiopia and other countries. The Pb concentration in this study was lower than that in agricultural soil in China and central Ethiopia [55, 56], and road dust in Pakistan [48]. While it was higher than the farmlands of Egypt [44], the levels of Co and Fe in the agricultural soil for the present study were lower than that of Iraq [57, 58] and Ethiopia (at East Gojjam Zone farmland) [30]. Moreover, the levels of Cd in soil samples in this study were lower than that around the Eastern Industrial Zone in Dukem, Ethiopia [31] and it was higher than that of the industrial areas of Bangladesh [59] and Ethiopia (at East Gojjam Zone farmland) [30]. Similarly, the mean level of Cr found in agricultural soils in this study was higher than that of Pakistan [34], the town of Debre Markos [60], and Bangladesh [59]. The Cu concentration in the agricultural soils was lower than that in Italy [61], Brazil [62], and Ethiopia (at Debre Work) [30]. Likewise, the mean value of Mn was higher than the values obtained for the farmlands of Indonesia [63] but was lower than the values obtained for Ethiopia (at the Mojo area) [58]. Generally, the levels of all analyzed elements in this investigation differ from other literature findings. The possible reason for this variation might be due to soil type, parent rock, climatic and topographical variation, and types and amounts of fertilizers and pesticides utilized by farmers [53, 64–66]. For instance, the levels of Pb, Cd, Cu, Fe, Mn, Ni, and Zn for the soil irrigated with Nile water were lower than the soil irrigated with wastewater [67]. Similarly, the level of Pb in the industrial zone of Iran was found to be higher than in the present study [68].

The mean concentration of PTEs in lentil crops varied from 60.4 (Fe) to 0.250 (Cd) mg/kg with increasing order of Cd < Co < Pb < Cr < Cu < Mn < Fe. The result in Table 1 showed that all investigated PTEs (except Cd) in lentil samples were found to be lower than the WHO/FAO permissive limits that the bioaccumulation of these PTEs seems to favor [69–71]. Furthermore, the levels of Pb and Fe in lentil samples in the present study were lower than those of the same sample in north Shewa [36], east Gojjam, and east Shewa, Ethiopia [72], despite the mean level of Co and Cu being higher in these areas [36]. The levels of Cu obtained in this study are also higher than those obtained from peas in Nigeria [73]. The levels of Cd and Cr in the lentil sample also showed higher levels than those reported in north Shewa [36] and lower than those reported in east Gojjam and East Shewa, Ethiopia [72]. The general concentration variation of all the analyzed elements in this investigation from other reports in the literature might be attributed to soil pH, organic matter type and distribution, plant age, crop type, micronutrient and macronutrient availabilities, and plant genotypes which vary from region to region [15, 74]. For example, the level of PTEs in tomato shoots and roots planted in contaminated farms was higher than in uncontaminated farms [75]. Similarly, the level of investigated PTEs in this study (lentil sample) was higher than that of the red grape samples grown in Gonabad vineyards [76].

### 3.2. Ecological Risk Assessments

The extent of pollution and risk of an ecosystem can be estimated by using single and cumulative impact indicators as seen in Table 2 [77]. The mean *I*_geo_, *C*_fi_, and *E*_*r*_^*i*^ values are descending in order of Cd > Cr > Cu > Fe > Co > Pb > Mn, Cd > Cu > Cr > Fe > Co > Pb > Mn, and Cd > Cu > Cr > Co > Fe > Mn > Pb, respectively. Cd's mean value is the highest indicating that it had a high accumulation in the soil. The mean value of Igeo for Mn, Fe, and Co were below 1, indicating low pollution, while the mean value of Igeo for Cu, Cr, and Cd indicates that these metals moderately polluted the soil. The single risk index parameter showed that the farmland was extremely contaminated by Cd metal despite no contamination being observed by other investigated metals. According to the contamination factor parameter result, the extent of farmland pollution was classified as low polluted by Mn, Pb and Co; moderately polluted by Fe, Cu, and Cr; and highly polluted by the Cd metal. Generally, a high level of single metal indicates high accumulation in the soil [53].

The cumulative impact of PTEs on the extent of soil pollution was estimated by using CD, mCd, PLI, and PINemerow. PINemerow and CD parameters indicate that the soil is highly contaminated by investigated metals, however, mCd and PLI showed moderate and no contamination, respectively. Overall, the risk to the ecosystem was evaluated by the RI parameter that indicated extreme toxicity [39]. Moreover, the enrichment factor result revealed that Cu and Cr showed minor enrichment, while Cd metal showed severe enrichment. A similar study in the study area district also showed a high amount of Cd [52].

### 3.3. Transfer Factor

The TF values of Mn, Fe, Co, Cu, Cr, Pb, and Cd were found to be 0.488, 0.093, 0.226, 0.142, 0.017, 0.493, and 0.016, respectively. Thus, the lowest and the highest TF values were observed in Cd (0.016) and Pb (0.493), respectively. The transfer factors of all PTEs were below 1, implying that the metal transfer from the soils to the plant was relatively low [78]. In contrast, the lowest level of TF in Cd metal, its amount in the crop, resulted in high bioaccumulation that was above WHO guidelines. The higher level of Cd bioaccumulation in the crop might be due to its high bioavailability in the soil [79–81].

### 3.4. Health Risk Analysis

#### 3.4.1. Daily Intake Dose (ADD)

The health risks (cancer and noncancer) of the local inhabitants were assessed as per the USEPA protocol, where HQ, HI, CR, and TCR were calculated based on the chronic daily intake of the PTEs through dietary (lentil crop) and nondietary (soil particle) sources [70, 82, 83]. The result of the average daily intake of different pathways of the investigated PTEs in the soil and lentil samples is given in Table 3. As can be seen in Table 3, the total intake of PTEs (mg/kg/day) by adults and children, regardless of the type of PTEs, were in the range of 0.0031 (from lentil)–8.93*E* − 08 (soil particle through inhalation) and 0.0106 (from lentil)–2.78*E* − 08 (soil particle through inhalation), respectively. The total daily intake of PTEs through any pathways from lentil and soil samples for adults was found in the range of 1.11*E* − 05 (Pb)–0.0021 (Fe) with the rank order of Pb < Co < Cd < Mn < Cu < Cr < Fe. A similar rank order of ADD_T_ was observed for children, with values ranging between 3.33*E* − 05 (Pb) and 0.0099 (Fe). The ADD_T_ values were highest for oral ingestion in adults and children, and the ingestion of soil particles appeared to be the main exposure pathway for potential toxic metals. Dermal contact and inhalation were the second and third most likely pathways. This result is comparable to similar studies [9, 84].

#### 3.4.2. Noncancer Risk Assessments

The potential noncarcinogenic toxic effects posed by single and multiple toxic metals are usually characterized by calculating HQ and HI based on different scenarios (Table 4). These scenarios are (1) single metal impact in each exposure pathway and sample type using HQ, (2) multiple metals impact across each exposure pathway using HI, (3) multiple metals impact in each sample type using HI, and (4) multiple metals impact in combined samples using HI.

The noncancer health risks of the present study related to individual element exposure through soil ingestion, soil dermal contact, soil inhalation, and lentil ingestion for adults and children were low for all the investigated metals, and the HQ was less than the unit [9]. Among the quantifying metals in the soil sample, the highest and the lowest HQ values were obtained in Cr and Co metals, respectively. The HQ value for each metal followed a similar trend in adults and children Pb < Cu < Cd < Co < Mn < Cr < Fe for soil particle inhalation, Co < Cu < Pb < Fe < Mn < Cr < Cd for dermal contact of soil particle, Co < Mn < Pb < Fe < Cu < Cd < Cr for ingestion of soil particle and Co < Fe < Cu < Mn < Cd < Pb < Cr for lentil ingestion. Cr and Cd are the most significant contributors to the total noncarcinogenic risk of adults, which accounts for 48.9 and 46.9%, respectively. Moreover, Cr and Cd account for 54 and 38.9% of total noncancer risk for children.

The multiple metal impacts also showed a low level of risk (HI < 1) in all scenarios for both adults and children (Table 4). Thus, the adult HI value for soil and lentil samples combined ranged from 0.0006 (Co) to 0.131 (Cr) with the order of Cox< Pb < Cu < Mn < Fe < Cd < Cr, while that of children were from 0.003 (Co) to 0.362 (Cr) with the order of Co < Mn < Pb < Fe < Cu < Cd < Cr. The different pathways that contributed to the noncancer risk follow dermal contact > ingestion rate > inhalation for both adults and children. The HI_len_ dietary intake routes were 39–73 order magnitude higher than the HI of soil in three exposure routes. The overall HI values for adults and children due to the intake of all investigated metals by all pathways from all samples were 0.268 and 0.668, respectively. Thus, the total HI value for children was approximately 3 times more than that for adults despite all results showing the low chance of noncancer risk for both inhabitants. Considering the total exposure of ingestion, dermal contact, and inhalation, there was a low chance of having noncancerous risk for adults' and children's health at the studied sites.

#### 3.4.3. Cancer Risk Assessments

As can be seen in Table 4, the cancer risk for the three carcinogenic metals (Pb, Cd, and Cr) was assessed based on their exposure to soil and lentil samples by following similar scenarios used in noncancer risk assessment. The carcinogenic risks from carcinogenic metals for adults and children via ingestion, dermal contact, and inhalation were within the acceptable ranges as established by USEPA (10^−4^ to 10^−6^), except Pb, which is below the guidelines (<10^−6^) [9, 70]. Compared to children and adults, adults are more seriously affected than children due to the high exposure time, and the result is in line with the study [9]. This study's CR and TCR results indicated no significant cancer development in adults and children throughout their lifetime. Moreover, among the three exposure pathways, soil ingestion is the primary pathway of exposure to hazardous elements, followed by dermal contact and inhalation. Therefore, the risk assessment of the present study provided baseline information for stakeholders regarding the potential risks associated with PTEs.

### 3.5. Statistical Analyses

The degree of correlation between the PTEs was performed using the Pearson's correlation statistical method. Thus, the correlation coefficient (*r*) value has been used to classify a given correlation as strongly correlated (*r* > 0.7), moderately correlated (0.5 < *r* < 0.7), and no correlation (*r* = 0). Moreover, a strong correlation also provides information about the sources of metals [9]. As can be seen from Table 5, a strong positive correlation between Cu and Fe, Pb with Cr and Co, and Mn with Fe and Cu in the lentil sample was observed. Similarly, Pb with Fe, Mn with Fe, Cr, and Pb, and Cd with Cr in the soil sample also showed a strong positive correlation. Moderate positive correlations were observed in Co with Cr and Cd in lentils and Cr with Fe and Cd with Cu in the soil samples. The PTEs with weak negative or positive correlation indicate that the presence or absence of one HM affects to a lesser extent than the other [47]. Overall, the more positive correlation between PTEs indicates that they had similar sources [53].

**Figure 2:**
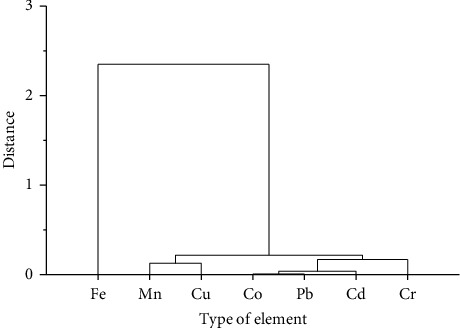
Dendrogram of the cluster analysis of seven elements in soil and lentil samples.

Moreover, a hierarchal cluster analysis (HCA) statistical test was employed for source identification of the investigated metals, and four clusters were obtained at a distance of about 0.3. Thus, Fe was grouped in cluster 1, Mn and Cu were grouped in cluster 2, Cd, Pb, and Co was grouped in cluster 3, and Cr in cluster 4. From the dendrograms, it can be concluded that Mn-Cu and Cd with Co and Pb have strong correlations suggesting that they have similar sources/origins. The details of the HCA are given as a dendrogram in Figure 2.

Generally, the present study has some limitations, including, not showing a special variation across each village in Dawunt Woreda, single-time sampling (could not show the temporary variation of PTEs), no analysis of pollutants other than PTEs (such as polyaromatic hydrocarbons), not including other crops and vegetables grown in the area, and the inability to identify most of the affected crops. Thus, other PTEs and toxic pollutants other than those included in this work are strongly advised in order to offer full data on the metal and other dangerous pollutants profile of the lentil crop and soil. Furthermore, PTEs' toxicity was influenced by their bioavailability, which was influenced further by the physical and chemical makeup of soils. As a result, additional examination of the soil's physical and chemical composition is strongly advised.

## 4. Conclusion

The levels of Fe, Mn, Cu, Co, Cr, Cd, and Pb in lentil crops and soil were quantified. The mean concentrations of these PTEs in the lentil and soil samples ranged from 60.4 (Fe) to 0.25 (Cd) and 649 (Fe) to 1.33 (Pb) mg/kg with the order of Cd < Co < Pb < Cr < Cu < Mn < Fe and Pb < Co < Cd < Mn < Cu < Cr < Fe, respectively. Apart from Cd in the soil sample, the levels of all investigated PTEs in lentil and soil samples were found below the standard limit value set by WHO/FAO. The single and multiple metal pollution indices parameters confirmed that the extent of soil contamination varied from moderate to high. The human health risk assessment revealed that there are no significant noncarcinogenic and carcinogenic risks for adults and children. This might be due to too low bioavailability of metals, as observed in lentil crops by transfer factor (TF < 1 for all metals). Overall, the pollution indices parameter showing the degree of soil pollution is classified as moderate to high. Thus, the inhabitants are likely to exhibit health risks to Cd, so much attention should be paid to Cd metal. Furthermore, the contribution of other metals other than Cd metals to future health impact is not small. As a result, a regular consumption of the lentil crop and spending a long time on the farmland might saddle inhabitants' health.

## Figures and Tables

**Table 1:** The mean, minimum, and maximum concentration of PTEs in soil and lentil samples (mg/kg) along with the recommended level of WHO/FAO.

Sample type		Fe	Mn	Co	Cu	Cd	Pb	Cr
Lentil	Minimum	59.4	9.530	0.700	5.660	0.240	0.840	1.13
	Maximum	60.9	9.770	0.790	5.740	0.260	0.990	1.18
	Mean	60.4	9.683	0.750	5.697	0.250	0.903	1.15
	SD	0.866	0.133	0.046	0.040	0.010	0.078	0.026
	WHO/FAO^*∗*^	425	25.00	8.00	10.0	0.100	1.500	1.30

Corresponding soil	Minimum	645	19.50	3.250	40.00	15.05	1.800	67.8
	Maximum	653	20.20	3.400	40.04	15.22	1.850	71.3
	Mean	649	19.90	3.317	40.02	15.15	1.833	69.06
	SD	3.73	0.350	0.076	0.021	0.091	0.029	1.933
	WHO/FAO^*∗*^	50000	2000	50	100	3	100	100
	Background^*∗∗*^	500	418	6.9	14	1.1	25	42

^*∗*^Permissible limits of FAO/WHO (1996). ^*∗∗*^The reference value (world average background values) used for ecological risk calculations.

**Table 2:** The different ecological risk indicator parameters.

Parameters	Fe	Cu	Mn	Pb	Cr	Cd	Co
*I*_geo_	−0.208	0.93	−4.981	−4.355	0.132	3.199	−1.642
*C*_*fi*_	1.299	2.859	0.047	0.0733	1.644	13.78	0.481
*E*_*r*_^*i*^	1.299	14.29	0.475	0.367	3.289	413	2.404
EF	1.00	2.20	0.037	0.056	1.266	10.67	0.370

**Table 3:** Average daily intake of PTEs from soil and lentils through inhalation, ingestion, and dermal contact exposure pathways.

Adults									
	Soil sample						Lentil sample		SL
PTEs	ADD_inh_‐can	ADD_inh_‐non	ADD_der_‐can	ADD_der_‐non	ADD_ing_‐can	ADD_ing_‐non	ADD_ing_‐can	ADD_ing_‐non	ADD_*T*_

Fe	7.26*E* − 08	1.57*E* − 07	1.97*E* − 05	4.27*E* − 05	0.000494	0.00107	0.000138	0.000299	0.002062
Cu	4.47*E* − 09	9.7*E* − 09	1.21*E* − 06	2.63*E* − 06	3.04*E* − 05	6.59*E* − 05	1.3*E* − 05	2.82*E* − 05	0.000141
Mn	2.22*E* − 09	4.81*E* − 09	6.02*E* − 07	1.3*E* − 06	1.51*E* − 05	3.27*E* − 05	2.21*E* − 05	4.79*E* − 05	0.00012
Pb	2.05*E* − 10	4.44*E* − 10	5.56*E* − 08	1.2*E* − 07	1.39*E* − 06	3.02*E* − 06	2.06*E* − 06	4.46*E* − 06	1.11*E* − 05
Cr	7.72*E* − 09	1.67*E* − 08	2.1*E* − 06	4.54*E* − 06	5.25*E* − 05	0.000114	2.62*E* − 06	5.68*E* − 06	0.000181
Cd	1.69*E* − 09	3.67*E* − 09	4.6*E* − 07	9.96*E* − 07	1.15*E* − 05	2.5*E* − 05	5.7*E* − 07	1.24*E* − 06	3.98*E* − 05
Co	3.71*E* − 10	8.04*E* − 10	1.01*E* − 07	2.18*E* − 07	2.52*E* − 06	5.46*E* − 06	1.71*E* − 06	3.71*E* − 06	1.37*E* − 05
ADD_*T*_	8.93*E* − 08	1.93*E* − 07	2.42*E* − 05	5.25*E* − 05	0.000607	0.001316	0.00018	0.00039	0.002569

Children									

Fe	2.23*E* − 08	2.42*E* − 07	2.24*E* − 06	2.42*E* − 05	0.000799	0.008658	3.72*E* − 05	0.000403	0.009924
Cu	1.38*E* − 09	1.49*E* − 08	1.38*E* − 07	1.49*E* − 06	4.93*E* − 05	0.000534	3.51*E* − 06	3.8*E* − 05	0.000626
Mn	6.83*E* − 10	7.4*E* − 09	6.84*E* − 08	7.41*E* − 07	2.44*E* − 05	0.000265	5.96*E* − 06	6.46*E* − 05	0.00036
Pb	6.3*E* − 11	6.83*E* − 10	6.32*E* − 09	6.84*E* − 08	2.26*E* − 06	2.44*E* − 05	5.56*E* − 07	6.02*E* − 06	3.33*E* − 05
Cr	2.37*E* − 09	2.57*E* − 08	2.38*E* − 07	2.58*E* − 06	8.5*E* − 05	0.000921	7.08*E* − 07	7.67*E* − 06	0.001017
Cd	5.21*E* − 10	5.65*E* − 09	5.22*E* − 08	5.66*E* − 07	1.86*E* − 05	0.000202	1.54*E* − 07	1.67*E* − 06	0.000223
Co	1.14*E* − 10	1.24*E* − 09	1.14*E* − 08	1.24*E* − 07	4.08*E* − 06	4.42*E* − 05	4.62*E* − 07	0.000005	5.39*E* − 05
ADD_*T*_	2.75*E* − 08	2.98*E* − 07	2.75*E* − 06	2.98*E* − 05	0.000983	0.010648	4.85*E* − 05	0.000526	0.012238

ADD_*T*_, the total average daily intake of all PTEs in each exposure pathway; ADD-non, the average daily intake for noncancer risk; ADD-can, the average daily intake for cancer risk assessment; SL, the soil and lentil sample combination.

**Table 4:** The values of cancer and noncancer risk indicator parameters for adults and children.

Noncancer risk										
	Adult					Children				
	Soil			Lentil	SL	Soil			Lentil	SL
PTEs	HQ_inh_	HQ_derm_	HQ_ing_	HQ_len_	HI^*∗*^	HQ_inh_	HQ_derm_	HQ_ing_	HQ_len_	HI^*∗*^

Fe	0.000715	0.00061	0.001274	0.000355	0.002954	0.0011	0.000346	0.010308	0.000479	0.012233
Cu	2.41*E* − 07	0.000219	0.001648	0.000704	0.002572	3.71*E* − 07	0.000125	0.01334	0.00095	0.014414
Mn	0.000336	0.000709	0.000696	0.001018	0.002759	0.000517	0.000403	0.005631	0.001373	0.007925
Pb	1.26*E* − 07	0.00023	0.000863	0.001275	0.002368	1.94*E* − 07	0.00013	0.006983	0.00172	0.008833
Cr	0.000585	0.090791	0.037924	0.001895	0.131194	0.0009	0.051565	0.306933	0.002556	0.361953
Cd	3.67*E* − 06	0.099605	0.024964	0.001236	0.125808	5.65*E* − 06	0.056571	0.20204	0.001667	0.260284
Co	0.000141	1.36*E* − 05	0.000273	0.000185	0.000613	0.000216	7.74*E* − 06	0.002211	0.00025	0.002685
HI	0.001781	0.192177	0.067642	0.006668	**0.268268**	0.002739	0.109148	0.547446	0.008995	**0.668328**

Cancer risk										
PTEs	CR_inh_	CR_derm_	CR_ing_	CR_len_	TCR^*∗*^	CR_inh_	CR_derm_	CR_ing_	CR_len_	TCR^*∗*^

Pb	1.64*E* − 14	1.56*E* − 08	3.9*E* − 07	5.77*E* − 07	9.83*E* − 07	6.3*E* − 16	1.77*E* − 09	6.32*E* − 07	1.56*E* − 07	7.89*E* − 07
Cr	9.27*E* − 11	4.19*E* − 05	2.63*E* − 05	1.31*E* − 06	6.95*E* − 05	1.99*E* − 10	4.76*E* − 06	4.25*E* − 05	3.54*E* − 07	4.76*E* − 05
Cd	3.05*E* − 12	1.18*E* − 05	7.37*E* − 06	3.65*E* − 07	1.95*E* − 05	9.38*E* − 13	1.34*E* − 06	1.19*E* − 05	9.85*E* − 08	1.34*E* − 05
TCR	9.57*E* − 11	5.37*E* − 05	3.4*E* − 05	2.25*E* − 06	**9E − 05**	2*E − *10	6.1*E* − 06	5.51*E* − 05	6.08*E* − 07	**6.18E − 05**

The bold font indicates the overall values of HI and TCR. HQ_inh_, hazard quotient for inhalation; HQ_derm_, hazard quotient for dermal contact; HQ_ing_, hazard quotient for ingestion; HQ_len_, hazard quotient for lentil ingestion; CR_inh_, hazard quotient for inhalation; CR_derm_, hazard quotient for dermal contact; CR_ing_, hazard quotient for ingestion; CR_len,_ hazard quotient for lentil ingestion; HI^*∗*^, hazard index for each metal from soil and lentil sample; HI, hazard index for all metal in each exposure route; TCR^*∗*^, hazard index for each metal from soil and lentil sample; TCR, hazard index for all metal in each exposure route.

**Table 5:** Pearson correlation of PTEs in lentil and soil samples.

	Lentil sample						
	Fe	Cr	Co	Cu	Cd	Pb	Mn

Fe	1						
Cr	−0.982	1					
Co	−0.756	0.619	1				
Cu	0.786	−0.655	−0.999^*∗*^	1			
Cd	0.00	−0.189	0.655	−0.619	1		
Pb	−0.918	0.826	0.954	−0.967	0.397	1	
Mn	0.997^*∗*^	−0.993	−0.705	0.737	0.075	−0.885	1

	Soil sample						

Fe	1						
Cr	0.659	1					
Co	−0.99^*∗*^	−0.694	1				
Cu	−0.86	−0.182	0.834	1			
Cd	−0.034	0.73	−0.014	0.539	1		
Pb	0.96	0.42	−0.945	−0.969	−0.314	1	
Mn	0.972^*∗*^	0.818	−0.982	−0.715	0.203	0.866	1

^*∗*^Indicates a significant correlation at a *p* value of less than 0.05 (two-tailed).

## Data Availability

The data used to support the findings of this study are included within the article.

## Supplementary Materials

Supplementary Materials Table S1: correlation coefficients and equations of the calibration curves for each investigated metal. The calibration of ICP-OES has been performed through a series of standard solutions. Thus, the linearity was performed by the coefficient of correlation (*R*^2^) of the calibration curve from one set of experimental measurements. Table S2: the values of instrumental detection limit (IDL), method detection limit (MDL), and limit of quantification (LOQ). The limit of quantification (LOQ) is the lowest concentration of an analyte in a sample which can be quantitatively determined with acceptable uncertainty. LOQ was obtained from triplicate analysis of seven method blanks which were digested with the same digestion procedure as the actual samples. Table S3: recovery test of soil and lentil samples. The accuracy of the instrument has been carried out using a standard addition method and by calculating percent recovery.
