# Supplementary material for: Potential Toxic Metal Concentration and Risk Assessment in Agricultural Soil and Lentil Crop (Lens culinaris Medik) in Dawunt Woreda, Northwest Wollo, Ethiopia
Source: J Toxicol. 2024 Apr 12;2024:8985402. doi: 10.1155/2024/8985402 (PMC11324366; doi:10.1155/2024/8985402)
Supplement: Supplementary Materials — Table S1: correlation coefficients and equations of the calibration curves for each investigated metal. The calibration of ICP-OES has been performed through a series of standard solutions. Thus, the linearity was performed by the coefficient of correlation (R2) of the calibration curve from one set of experimental measurements. Table S2: the values of instrumental detection limit (IDL), method detection limit (MDL), and limit of quantification (LOQ). The limit of quantification (LOQ) is the lowest concentration of an analyte in a sample which can be quantitatively determined with acceptable uncertainty. LOQ was obtained from triplicate analysis of seven method blanks which were digested with the same digestion procedure as the actual samples. Table S3: recovery test of soil and lentil samples. The accuracy of the instrument has been carried out using a standard addition method and by calculating percent recovery. [file 8985402.f1.docx]

TABLE S1: Correlation coefficients and equations of the calibration curves.

| Heavy metals | Amount of Standards (mg/L) | Correlation coefficient (R^2^) | Calibration equation |
| --- | --- | --- | --- |
| Cu | 0.5,1.0,1.5,2,2.5,3.0 | 0.9994 | y = 3946x – 174 |
| Mn | 0.5,1.0,1.5,2,2.5,3.0 | 0.9995 | y = 37553x + 193 |
| Cr | 0.2,0.4,0.6,0.8,1.0,1.2,1.4,1.6 | 0.9967 | y = 66518x – 111 |
| Fe | 1,2,3,4,5,6 | 0.9967 | y = 10400x + 780 |
| Co | 0.5,1.0,1.5,2,2.5,3.0 | 0.9945 | y = 9379x – 328 |
| Cd | 0.2,0.4,0.6,0.8,1.0,1.2,1.4,1.6 | 0.9968 | Y = 16819x - 257 |
| Pb | 0.2,0.4,0.6,0.8,1.0,1.2,1.4,1.6 | 0.9958 | Y = 901.5x + 3.09 |

TABLE S2: The values of instrumental detection limit (IDL), method detection limit (MDL) and limit of quantification (LOQ)

| Heavy metal | IDL | MDL | LOQ |
| --- | --- | --- | --- |
| Cu | 0.001 | 0.003 | 0.01 |
| Fe | 0.002 | 0.171 | 0.57 |
| Mn | 0.002 | 0.009 | 0.03 |
| Cd | 0.001 | 0.003 | 0.01 |
| Co | 0.007 | 0.009 | 0.01 |
| Cr | 0.001 | 0.006 | 0.02 |
| Pb | 0.002 | 0.006 | 0.02 |

$LOD={3 x SD}_{b}$ (i)

$LOQ={10 x SD}_{b}$ (ii)

Where SD_b_ is the standard deviation of the method blank

TABLE S3: Recovery test of soil and lentil samples.

|  | Soil sample | | | |  |
| --- | --- | --- | --- | --- | --- |
| HMs | Un-spiked Amount (mg/kg) | Amount added in (mg/kg) | Amount after spiked (mg/kg) | %Recovery | %RSD |
| Mn | 19.850±0.35 | 7.94 | 27.28±1.967 | 93 | 1.76 |
| Fe | 649.38±3.73 | 259.7 | 1864.9±6.10 | 83 | 0.57 |
| Co | 3.317±0.076 | 1.32 | 4.6±0.229 | 98 | 2.29 |
| Cu | 40.020±0.018 | 16 | 54.4±4.534 | 90 | 0.05 |
| Cd | 15.160±0.093 | 6 | 20.26±0.135 | 90 | 0.6 |
| Pb | 1.833±0.029 | 0.7 | 2.5±1.409 | 100 | 1.58 |
| Cr | 69.063±1.931 | 27.6 | 92.76±0.368 | 86 | 2.80 |
|  | Lentil sample | | | |  |
| Mn | 9.686±0.013 | 3.8 | 13.4±0.130 | 98 | 1.37 |
| Fe | 60.4±0.09 | 24.16 | 81.66±0.31 | 88 | 1.43 |
| Co | 0.75±0.005 | 2 | 19.84±0.016 | 95 | 6.13 |
| Cu | 5.70±0.004 | 4 | 40.62±0.053 | 87 | 0.70 |
| Cd | 0.25±0.004 | 2 | 19.17±0.008 | 94 | 4.00 |
| Pb | 0.35±0.002 | 2 | 19.48±0.027 | 95 | 8.64 |
| Cr | 1.15±0.003 | 1.5 | 15.12±0.012 | 93 | 2.26 |

$\% Recovery= \frac{Amount in spiked sample - Amount in unspiked sample}{Amount added.}$x 100 (iii)

$\%RSD= \frac{SD}{Mean}x 100$ (iv)

Where SD is the standard deviation of each metal in each sample.
